# How Public Home Care Officers Reason When Making a Needs Assessment for Food Distribution to Homebound Elderly Persons in Sweden

**DOI:** 10.5539/gjhs.v5n5p31

**Published:** 2013-05-22

**Authors:** Zada Pajalic

**Affiliations:** 1 The School of Health and Society, the PROCARE Group & The Network for Eating and Nutrition, Kristianstad University, Sweden & The Action Learning, Action Research Association (ALARA), Australia

**Keywords:** elderly, food distribution, homebound, grounded theory, public administration

## Abstract

Food distribution (FD) is a part of the public social and care service in Sweden aiming to prevent improper food intake for persons that they are unable to do their own shopping, and prepare their own meals, and in that way ensure reasonable standard of living. Before a person can be granted the FD service, from any municipality, an assessment of their individual requirements has to be made by a public home care officer.

The aim of this study was to explore how public home care officers reason when they make a needs assessment for homebound elderly people.

The data was collected through individual interviews (n=18). The transcribed interview material was analysed by means of the grounded theory method.

The findings showed that the public home care officers were confronted with many challenges when making an assessment of a person's individual needs. They are influenced by their subjective feelings related to their personal views as to what should be the right solution for the individual. However, they remained aware that they needed to be guided by the legal requirements. Further, they described that the level of an individual's living standard is a leading concept in the governing laws that they need to interpret. Interpretation of this concept is very subjective with the possible consequence that an assessment result may lead to inefficient support.

In conclusion, the concept of a reasonable standard of living needs to be clearly defined, decision regarding FD should not take long time, need assessment and decision should be based on the whole picture behind each individual case and there are needs to develop general guidelines for making needs assessment. The findings in this study have implications for public administration, nursing and gerontology.

## 1. Background

Food distribution (FD) is a part of the public social and care service in Sweden aiming to prevent improper food intake for persons that they are unable to do their own shopping, and prepare their own meals, and in that way ensure “*reasonable standard of living*” (Pajalic, Westergren, Skovdahl, & Persson, 2013). The concept *reasonable standard of living* should be a guaranteed minimum level, based on the client's circumstances. The concept is not defined in law texts in a general way which gives the possibility for each public home officer to interpret the concept in their own way (Socialstyrelsen, 2011).

All the public social and care service in Sweden is a statutory obligation for society and regulated by two acts: The Health and Medical Service Act and The Social Service Act (Grönwall & Holgersson, 2000; Raadu, 2011).

The municipalities (n=290) have the responsibility for the FD. The fundamental requirement for a person being granted FD is that they are unable to do their own shopping and prepare their own meals, and that the foremost reasons for this situation are illness related physical or psychical limitations. Only when elderly people are not able to make food by themselves and their needs cannot be met in any other way, does it become a public, i.e. municipal, obligation. Many elderly persons are disabled and dependent on others for acquiring and preparing food. Approximately 200 out of a total of 290 municipalities in Sweden (a population of 9 256 347 people) produce food for FD, most often in the form of lunch, either distributed daily as a hot meal (60 degrees) or weekly as a cold meal. The possibilities to have evening meals delivered exist. The number of elderly persons in Sweden receiving food by FD is estimated to 60,000, and the population prognosis shows that the need for FD will increase in future (SCB, 2009).

Before a person can be granted the FD service, from any municipality, an assessment of their individual requirements has to be made by a public home care officer (Pajalic, Persson, Skovdahl, & Westergren, 2012). Public home care officers in Sweden have an administrative position within the municipality, dealing, among other things, with applications for assistance. A Swedish public home care officer most often has a degree in social work with a specialisation in specific assessment and legal regulations (Thorslund & Wånell, 2006). Further, the public home care officer is expected to make a judgment on the assessment material, on behalf of the municipality, in accordance with the current legislation, guidelines and conditions (Clow, 1995).

The procedure for assessment of elderly people's needs may be initiated in different ways e.g. a direct request from the person who needs help, from relatives or from health care personnel with whom the person has been in contact with at the in and outpatient care (Pajalic, Persson, Westergren, Berggren, & Skovdahl, 2012). If the initiative for a request for an assessment comes from someone other than the elderly person requiring help, it is very important that the that the elderly person concerned agrees with the initiative and formally submits a personal application (Pajalic et al., 2012). Once the application is submitted the public home care officer will begin the assessment and, based on the results, determine whether to grant or deny the application (Grönwall & Holgersson, 2000; Raadu, 2011). The organisation of public social and care service in Sweden often differs from other welfare states, primarily due to the fact that the public social and care service is based on the State's legal obligation to help needy persons and is financed by taxation (Elmér, 2000). In Sweden, the relatives of a person requiring any type of social assistance, including their children, have no specific legal obligations or care responsibility towards their next of kin. These matters are the responsibility of the state through the municipalities (Szebehely, 2003). The charges for public social and care services are regulated and based on the receiver's ability to pay. However all those covered by the act are obliged to pay a maximum of 10% of the total costs (Elmér, 2000).

However, a literature review showed that there is no existing research material focusing on how the public home care officers reason when they make a needs assessment for FD to homebound elderly persons. This knowledge is important in order to provide more information about the little researched issue of how public home care officers reason when they make this type of needs assessment.

The aim of this study was to explore the question: How do public home care officers reason when they make needs assessments for homebound elderly people?

## 2 Method

### 2.1 Context

The study was conducted within two average-sized municipalities, totaling about 130 000 inhabitants, and all situated in the southern part of Sweden. Approximately 850 elderly people, living in these municipalities, used the municipal FD service. This number could vary since some recipients only used the FD service on certain days of the week. The meals were produced at municipally owned kitchens. The menu consisted of two hot dishes each day and an extraordinary meal once a month. The most common meal delivered was lunch, which corresponded to 30% of the clients’ daily nutritional needs. The possibility to also have evening meals delivered did exist, but was not commonly used. The lunch also included raw vegetables and fruit or dessert all inclusive. Assistant nurses or taxi drivers were responsible for the transportation and delivery of the meals. The meal box was supposed to be delivered personally to the customer, that is to say “into their hands”, and not simply left outside their door. The FD is part of the Swedish welfare system and that means that people who have the need for help in obtaining ready prepared meals can retain this service. The procedure, after application, is that a public home care officer completes the necessary assessment and makes a decision on the applicant's need for the FD service. Their decision can be either to grant or deny the application. In case of denial, the public home care office is obliged to inform the applicant about how they can appeal for a new assessment.

### 2.2 Participants and Data Collection

The participants in this study were selected through purposeful sampling (Patton, 2002), the selecting criteria were that the participants should be involved in assessment of needs for FD. The data they provided was collected through individual interview with the public home care officers (n=18). Saturation was reached after 15 interviews and analyses, after which three additional interviews were conducted to ensure the quality of the information. All of the informants were women with between 3 and 20 years working experience as public home care officers. A pilot interview was conducted to test the validity of the questions. All the interviews took place at the informants’ work place. The questions (Table 1) were given to the participants at the start of the interview, which lasted between 30-90 minutes. The data was collected using tape-recorded interviews. All the interviews were transcribed verbatim and the analysis began simultaneously.

**Table 1:** Examples of questions used in group-interviews and individual interviews

Can you describe your first contact with the elderly person who applied for FD?
What is important to think about during this first contact?
What are your experiences related to the possibilities to meet the needs for those who apply for FD?
What can be problematic when it comes to granting or denying FD?
How do you follow up decisions for FD?
What aim do you have in your work with FD assessments?
Are there guidelines for the needs assessment for FD?
Does the municipality's economy influence yours possibilities to grant FD?
How much can the elderly people's wishes influence your decision?
Which considerations do you apply when an elderly person expresses a wish?

### 2.3 Data Analysis

The data was analysed using grounded theory (Charmaz, 2006; Glaser, 1978). The researcher listened first to the tapes, read through the transcripts to get feeling for the whole, extracted the facts and then applied a substantive coding technique. Substantive coding was performed in the following steps. Firstly the transcripts were coded line-by-line (*in vivo)* using the phrases used by the participants’. Then the phrases were shortened and developed into a code that captured the main idea of what the participant had said. Coded phrases were reduced by grouping them together into similar codes and phrases. Then the terms were evaluated into concepts. The next step was the naming of the categories followed by theoretical coding in order to isolate the concepts and connect them. This was followed by seeking new connections of categories and the definition of the subcategories. The emerging of defined categories was linked. The next step was the definition of the core category and the identification of the basic social process by the leading core category. Finally, an explanatory framework was created which led to the derivation and development of a substantive theory.

### 2.4 Ethical Considerations

The study was performed in accordance with the Helsinki Declaration (Saif, 2000), and has been examined by the Regional Ethical Review Board in Lund (LU09/365). All participants gave their informed consent to participate in the study after having been presented with detailed information about the study and their own participation. They were also informed that they had the right to terminate their participation at any time without it having any consequences for them.

## 3. Findings

### 3.1 The Derivation and Development of a Substantive Theory

The participants had quite similar experiences related to elderly people requesting a needs assessment for FD. They were confronted by many different types of individuals applying for FD and had a need for municipal support in the form of FD. The participants described how the initiative for applying for a needs assessment for FD usually comes from elderly persons, their relatives or the health authorities. The reasons for applying for FD are mostly based on physical, physiological or psychosocial problems. As the municipal FD service is tax supported it has to be officially applied for, giving the reasons behind the request which need to be of a required social support nature. It is not possible to receive the FD service simply for personal convenience. The application for FD may be made verbally or in writing. Once the application has been submitted the public home care officers plan a meeting with the applicant. If there is an obvious need to decide a request for FD, with immediate effect, the public home care officers can make an immediate decision after gathering the necessary assessment data by telephone. An FD need assessment focuses mostly on the applicants stated reasons for requesting FD. As one of the participants remarked:

*“FD is for those who have a legitimate need. Most of the* *elderly do not wish to have FD if they do not feel that they have to. Sometimes there are people who apply for FD because they are tired of preparing food and would rather focus their energy on other things, but this is not a legitimate reason for approval, however, the final decision is based on the reasons and facts derived from a needs assessment interview…*”

In some cases, the public home care officers judge that there is an apparent need to grant the FD service to an applicant, but find that the applicant has not or is unable to give all the information required for them to reach a positive decision. In such cases the officers will turn to the applicant's relatives or any involved health care personnel to complete the information they have.

Once a decision is made it is passed on to the applicant and the administrative managers responsible for the FD service. The administrative managers have the responsibility to execute the public home care officer's decision. The participants stressed that it is the administrative managers who have the actual responsibility for distributing the food even during an uncompleted, ongoing assessment process, as the applicant must have food in order to avoid the consequences of under-nourishment. Sometimes the decisions are made instantly, such as, in situations where an elderly person is about to be discharged from hospital.

The participants stressed that is important that no one should have to wait a long time for a decision regarding FD. The mealtime that is the most common consideration for their decisions is lunch. The applicant has the responsibility for all their other meals, such as breakfast, snacks and evening meal. If the public home care officers estimate that an elderly person has a further need for FD, they make the decision to continue FD. Also, if the public home care officer believes that an elderly person has a need for FD but that the final decision has not been made, the officer will grant FD until further notice.

The participants admitted that they do not usually make a new need assessment in these cases. In cases where an elderly person needs support during their mealtime, the public home care officers complement their decision with details regarding the requirements for specific assistance during mealtimes. It is foremost the home help personnel who observe the elderly person's needs while distributing the food, after which they pass on their observations to a public home care officer who then makes a decision for the specific case. However, what is to be done in practice is not their responsibility. For instance, they do not make the decision concerning how much time the additional service may take

But if there is a need for mealtime assistance they specify it in their decision. The public home care officers noted how the FD does not demand the involvement of medically educated personnel such as qualified district nurses and described the situation as follows:

“The need for advanced feeding by tube is a medical situation whereas eating with a knife and fork is not. Such help is an independent assistance form and does not demand the involvement of a district nurse. The eventual involvement of a district nurse is initiated by the primary care.”

The participants pointed out that they primarily look to the needs of an FD applicant rather than simply the cost of the service. Sometimes it happened that an elderly person declined the right to FD. In such cases the elderly person's decision is taken into consideration. The participants noted that they do not force anyone to accept municipal FD against their wish. In the situation where the public home care officers deny an application for FD, it is usually those applications from persons who can make food by themselves or can resolve their meal requirements in another way. One of the participants expressed it as follows:

“Wishing cannot be the basis for assistance.”

The participants maintained that they always look at the whole picture behind each individual case when they make their assessments and decisions. In situations where the public home care officers feel insecure about their decision making, they consult their colleagues or check with them to see if they had missed anything. The participants referred to concept of “a reasonable standard of living” that remains to be a subject for individual interpretation which can result in an inadequate assessment and result with rejection. The concept of a reasonable standard of living remains to be a subject for individual interpretation which can result in an inadequate assessment which may lead to inefficient support influence. The rejection can have a negative influence on an applicant's nourishment and quality of life.

**Figure 1:**
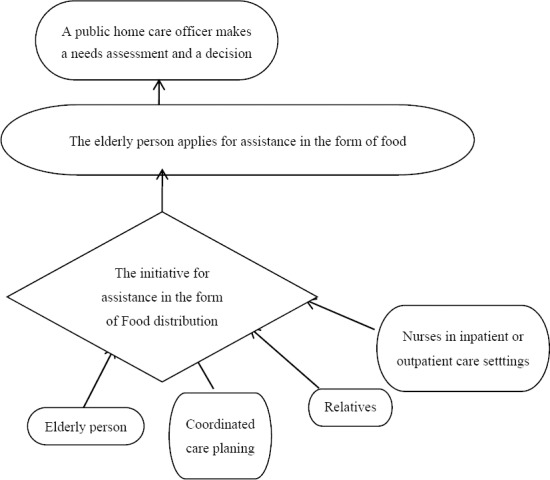
The flowchart showing process from initiative for FD assistance to decision

### 3.2 Application for FD

The participants described how their first contact with an elderly person in need of the FD service is often through the telephone or at the point of an elderly person's discharge from hospital. Following first contact, the home care officer requests to make a house call to the applicant. The most common reason for applying for FD is the applicants’ growing lack of energy, fatigue or forgetfulness to shop and make food. At the first assessment meeting the applicant is asked to explain their concerns related to their way of life and to describe what they can or cannot manage. Sometimes relatives are present at the meeting as support and to clarify the applicants concerns. The discussions at the meeting are followed by the offer of information about how the municipality can support the applicant. The participants described that they make their assessment regarding an applicant's need for FD based on the facts given in the application. The participants described how they offer written information concerning how the FD is organised and administered throughout the day. An assessment focuses mainly on the applicant's general condition both physically and psychologically, their social network, and whether or not the applicant can prepare their own food even though they have the necessary skills, Further consideration is given to whether or not the applicant can reasonably visit a municipal restaurant to have their meals. One of the participants described an FD application situation they had experienced:

“The applicant spoke about their concerns and what difficulties they experienced, such as what tasks they felt able to do themselves. Sometimes there are relatives present when we visit applicants. Usually we meet the elderly applicant in their home; we discuss with them how they deal with their mealtimes throughout the whole day. Through the interview I am able to reveal any preconditions and limitations the applicant may have that hinders them from arranging their own meals. This means assessing the applicant's general condition including their physical and psychological health, and also their contact network. At an interview it is also possible to discern whether the applicant could make their own food or were able to get to a day centre restaurant.”

### 3.3 Important to Take into Consideration

The participants stressed that it is important to take into consideration whether or not the elderly applicant can make their own meals or it they can fulfill their food needs in another way. Further they try to determine what the applicant can make by them self, if they need help to prepare food, if they can train to regain lost body functions and what type of assistance they need Further, it is also important to observe the elderly person's nutritional in-take throughout the day, the applicants whole life situation, and if it is possible that they can achieve reasonable living conditions. The participants stressed the importance of always having good communication with each individual applicant and, to not go over their head. They, as assessors, can only decide what the applicant has a clear need for.

Sometimes they need informed consent from the applicant to collect information from primary care centres and to send their decision to the applicants so that they can have insight into the decision. On completion of needs assessment their decision is sent to the applicant so that they may have an insight into the process.

The participants stressed the importance of confidentiality even where relatives are involved. They described how the applicant can choose to limit the scope for the distribution of their personal information and the home care officer's decision. This limitation also includes their children. Further the applicants have the possibility and right by law to update the data in their needs assessment, however, this seldom happens. One of participants illustrated this possible situation as follows:

“If I approve FD to an applicant then later on it is found that the applicant is able to prepare their own food, it becomes hard to motivate the continuation of the FD.”

### 3.4 Factors That Make it Difficult to Make an Assessment

The participants described some factors that make an assessment process difficult. As examples they noted: that there was no need for supplying FD if the elderly applicant was quite able to make their own food, that it was hard to evaluate if the applicant was able to get to a day centre restaurant for a meal. It is not easy to evaluate how much the elderly can do without help or, to evaluate when their need no longer exists. The participants stressed that an assessment seldom leads to a denial. One of the participants expressed it follows:

“How much can we actually demand?… On the one hand we can feel sympathy with the applicant; however this is not sufficient grounds for granting FD. It can feel hard, but it is part of our profession. In some case the applicant's relatives exaggerate and their observations are not always based on reality.”

### 3.5 Following up a Decision

The participants described how a granted FD application is followed up, usually after three weeks. The grant for FD is valid for one year after which, if there remains a case for granting a further year, a new assessment is made. The participants stressed that even the applicant can initiate a new assessment at any time.

“First, we contact the homecare personnel and then we phone the elderly person and ask how things are. If something is not right we ask the person if we may make a home visit.”

According to the participants their most important goals were to make their decision in accordance with the governing regulations, to achieve a reasonable standard of living for the applicant, to grant FD to those who have the need and right to the service, to keep the applicants informed and give them the possibility to appeal against an eventual denial of their application. Further, it is important that the applicants are satisfied with the service.

One of the participants expressed it as follows:

“What is considered to be a reasonable standard of living is not described in The Social Service Act. It is related to the applicant's needs. We interpret this from own frame of reference. Although we interpret the regulations ourselves we do so while bearing in mind that we have the responsibility to offer proper assistance and support to the citizens in our municipality. My interpretation of a reasonable standard of living is that no one shall live in poverty and that the services offered shall correspond to the requirements of those with a low income related to what is considered as the normal Swedish standard of living. It is a question of interpretation. However, to meet this situation, those elderly who have a low pension may receive FD free of charge.”

### 3.6 The Guiding Principles

The participants stressed that there are no general guidelines for making a needs assessment. All assessments are based on individual needs. One of the participants remarked that an important guideline for her is whether or not the elderly person, being assessed, can walk to the nearest day centre for their meals. One of them described her reasoning as follows:

“…It is The Social Service Act that guides my assessment and judgment when deciding on a person's right to FD. That is to say, if the applicant cannot prepare their own lunch and are unable to resolve the problem in any other way it becomes the municipality's responsibility to assist, for example through the FD service…”

### 3.7 The Influence of the Municipality's Economy on Decisions Related to Granting FD

The participants stated that the local politicians do not have a direct influence on their assessments but that the municipality's economy does. The basic principle is that a municipality's economy is based on taxes paid by local citizens. Further they stressed that the municipality is obliged to offer assistance to citizens who are in need. The participants noted that they have weekly meetings at their offices where they have the opportunity to discuss their cases with each other, especially those cases that are difficult. One of the participants expressed it as follows:

“The politicians influence our assessment through the application of fixed guidelines which we are obliged to follow, the guidelines are common for all municipalities and are decided by the politicians and updated each year, often there are small adjustments made, The Social Service Act steers our work.”

### 3.8 The Applicant's Possibility to Influence the Outcome

The participants stressed that they try to take into account the elderly person's wishes but it is the person's obvious need that steers any decision. All an applicant's wishes are handled within the framework of the Social Service Act. Further they described how the elderly have the possibility to choose the frequency of FD from every day each week to just some days a week. They also stressed that they take into account the elderly person's health status and their possible need for a special diet. They listen to their eventual wishes and determine if they are possible to fulfill. For instance, there are many applicants who wish for service in the form of having someone to cook for them at home, but this type of service is seldom granted. Help with cooking can be granted if it is part of a rehabilitation or training programme for persons with reduced bodily functions.

## 4. Discussion

### 4.1 Methodological Considerations

Findings from this study can be evaluated in terms of trustworthiness, credibility and dependability (Glaser, 1978; Lincoln, & Guba, 1985). Trustworthiness was used to ensure conscientiousness and honesty from the start of the research process, through the different steps of the analysis, in order to present a clear and complete picture finally through identifying statements from the participants, while, at the same time, a continuous comparison between data, codes and categories was made throughout the analysis (Charmaz, 2006; Fridlund, 1998). Credibility was assured by presenting views from different participants who were purposely chosen in order to capture different experiences. The purpose of research with grounded theory design is to achieve an understanding of social processes that explains the individual's actions in relation to others (Glaser, 1978). Further the credibility was ensured by that the sample of informants was representative and that the method of data collection was relevant to the aim of the study. As the participants themselves applied for participation in the study, the author was well aware of the possibility that this might have had an impact on the theory generated. There is reason to suspect that participants who themselves have chosen to apply for participation may deviate from the public home offices that did not participated in the study. Dependability was assured by the fact that the same researcher, the author ZP, carried out all the interviews, analysis and transcriptions. The use of a tape-recorder and verbatim transcripts, as well as referring back to, and re-reading the transcripts during the analysis process, allowed the researchers to remain close to the text. The citations make it possible to assure conformability. The authors pre-understanding was perceived as strength although there may be a risk of giving rise to biases. In order to minimize this risk as much as possible, the researcher attempted to be as open-minded as possible during the interviews. Control was ensured only after the implementation of a pilot interview, was reached through continuous movements back and forth between the whole and the parts during the analysis process as well as confirmed in the data.

### 4.2 Discussion of the Results

The study results showed that public home care officers use both subjectively and objectively based reasoning during a needs assessment process. The results in other study showed that follow-up decisions based on needs assessment were experienced as the last phase of need assessment (Janlöv, 2006). The routine was to offer help in accordance with the municipal guidelines based on the Social Service Act (Blomberg, 2004). The Public home care officer's responsibility covered even the organisation's general guiding principles and legislation (Wolmesjö, 2005). This also includes understanding the legislation and ensuring correct implementation, taking necessary precautions and observing the need for self-protection (Eloranta, Arve, & Routasalo, 2008). Boundaries and responsibility are related to personal experience, feelings and values. Further in Chevannes's (2000) study it was shown that the social construction of the managerialism and the justifications for the assessments made resulted in dominance, by the professionals, in the needs assessment process. He pointed to the importance of the elderly persons rights and their possibility to participate in decision making and to be treated as an equal part in the process (Chevannes, 2002). On the other hand, Larsson's (2006) study showed that legislation and the feeling of doing right in accordance with the law was related to a need for self-protection and the consideration of professional responsibility (Larsson, 2006). Legislation could be viewed as being power or a shield to hide behind. Another study pointed out that needs assessment should be seen as a professional responsibility to find the balance between demands and individual needs and an effort to gain for wholeness in accordance with ethical principles (Janlov, Hallberg, & Petersson, 2011).

The results in the present study showed that public home care officers preferred to have contact with the applicant's relatives in cases where the applicant could not convey the details of their disabilities and what it is they have a need for. In the other study was shown that such cases public home care officers are careful to ensure that the relatives understand that even in their case it is important that the applicant should be involved in the needs assessment (Hammar, Perälä, & Rissanen, 2009). Karlsson (2008) showed that public home care officers try to gain the whole picture when making a needs assessment; this includes the applicant's functional ability, health status, and their agreement to the needs assessment process and their satisfaction with the care they receive. Public home care officers focus more on creating trust when meeting an applicant and protecting the applicant's integrity. The assessment is made within the framework of social relations allowing negotiation and is basically socially constructed (Karlsson, 2008). The relatives often have a larger burden than they can master so the needs assessment demands the building up of the whole picture during the first meeting with the applicant and their relatives (Thorslund & Wånell, 2006). Meeting with everyone concerned is central to the public home care officer's approach, as it allows the creation of contact, relation preservation, and coordination, passing on of information, offering support and creating control over the situation. The meeting contributes towards gaining better insight and understanding of an applicant's situation. Further, meeting those involved gives the home care officer the opportunity to examine the trustworthiness of the application and to observe the applicant within their social environment (Lagergren et al., 2004). Further the public home care officer will use the opportunity to build their personal view of the value of what is expressed verbally and what has been written in the application.(Szebehely, 2006). The decision process focuses on a conceptualisation by the home care officer of the applicant's situation based on detailed evidence relevant for the application. The decision process is described as a process based on the public home care officers personal experiences adjusted to the individual applicant and the situation, but at the same time the decision process is steered by the legal demands that follow with the public home care officer's position in the organisation. The length of personal contact with an applicant can influence the applicant's possibility to influence their own situation (Nordström, 2000). It was important not to override an applicant's will (Janlov, Hallberg, & Petersson, 2006). The participation of the applicants relatives was experienced as positive, however, if they have the rolle of guardians, or ‘power of attorney’ they could be experienced as troublesome as normally elderly persons are experienced as being mostly prepared to accept their commune's proposals, while relatives often have additional demands (Hellström Muhli, 2003; Lindelöf, & Rönnbäck, 2004).

This study shows that public home care officers strive to build a complete picture of the applicants needs by meeting them. Further it was described how public home care officers involve the applicants’ relatives in cases where the applicant cannot participate fully due health related problems. The concept of a reasonable standard of living remains to be a subject for individual interpretation which can result in an inadequate assessment which may lead to inefficient support influence and in turn can have a negative on an applicant's quality of life related to nutrition. There are no general guidelines for making needs assessment. There is a need to clearly define, by law, the concept of a reasonable standard of living with the objective of preventing wide interpretation and inadequate assessment (Table 2).

**Table 2:** Key findings

• The concept of a reasonable standard of living needs to be clearly defined
• Decision regarding FD should not take long time
• Need assessment and decision should be based on the whole picture behind each individual case
• There are needs to develop general guidelines for making a needs assessment
